# *SOX9* gene anomalies and campomelic / acampomelic campomelic dysplasia: case report and literature review

**DOI:** 10.3389/fgene.2026.1755075

**Published:** 2026-03-10

**Authors:** Craig V. Towers, John T. Meadows, Peter T. Petruzzi, Kelsey L. Grabeel

**Affiliations:** 1 High Risk Obstetrical Consultants, University of Tennessee Medical Center, Knoxville, TN, United States; 2 Department of Neonatology, University of Tennessee Medical Center, Knoxville, TN, United States; 3 Department of Radiology, University of Tennessee Medical Center, Knoxville, TN, United States; 4 Preston Medical Library / Health Information Center, University of Tennessee Health Science Center College of Medicine, Knoxville / University of Tennessee Medical Center, Knoxville, TN, United States

**Keywords:** acampomelic campomelic dysplasia, disorders of sexual development, campomelic dysplasia, genetic counseling, Pierre Robin sequence, *SOX9* gene

## Abstract

Campomelic dysplasia (CD) is a rare skeletal disorder characterized by the hallmark sign of bent femur or tibial bones or both. Subsequently, patients were identified as having features of CD but lacking the bent limbs. This constellation was later described as acampomelic campomelic dysplasia (ACD). Both CD and ACD are caused by anomalies in the *SOX9* gene. Historically, CD has a high mortality rate, and ACD patients are often described similarly. Parents may be counseled to consider pregnancy termination or palliative care. This manuscript describes an index patient with ACD who exhibits prolonged survival, along with an extensive literature review. This review shows that roughly 9 out of 10 cases of acampomelic campomelic dysplasia with a genetic diagnosis survive beyond 1 year of age, most of whom are over 2 years old. In stark contrast, only approximately 3 out of 10 CD cases with a genetic diagnosis survive infancy. However, this CD number is skewed because survival beyond infancy is 2 in 10 or less for splice-site and nonsense pathologic variants, deletions, and insertions; approximately 4 in 10 cases for missense pathologic variants; and approximately 8 in 10 cases for chromosome 17 structural rearrangements. These findings are of critical importance for patient counseling and perinatal care planning.

## 1 Introduction

Campomelic dysplasia (CD) is a rare skeletal disorder characterized by the hallmark signs of bent femur or tibial bones or both. The early history of CD has been recounted by others, but some misinformation remains. In an excellent review, Angle (1954) identified 14 studies published between 1915 and 1952. These manuscripts described a total of 37 cases (38 including her own case) involving newborns with bent femur or tibial bones or both. Following this, additional publications described similar cases until October 1970, when Spranger, Langer, and Maroteaux reported in The Lancet on nine patients with similar findings, referring to it as “a syndrome of multiple osseous defects” (Spranger et al., 1974). In The Lancet on 15 May 1971, Bianchine et al. (1971) (Baltimore) published a case and called it “camptomelic dwarfism.” In The Lancet on 22 May 1971 (a week later), Maroteaux et al. (1971) (France) reported on other cases and called it “campomelic syndrome.” In 1977, at the Paris International Nomenclature meeting, this disorder was given the name “campomelic dysplasia” (International Nomenclature of Constitutional Diseases, 1978). Then, Lindgren and Ringertz (1980) published an abstract in 1979 that described a patient with “campomelic syndrome without campomelia.” Since then, this variant disorder has been named “acampomelic campomelic dysplasia” (ACD). The Online Mendelian Inheritance in Man (OMIM) number for CD is OMIM114290. ACD does not have its own OMIM number and currently falls under CD.

In the early 1990s, it was discovered that CD and ACD were caused by genetic abnormalities of the *SOX9* gene located on the long arm of chromosome 17. Over the past 30-plus years, chromosomal translocations and inversions involving chromosome 17; missense (MS), nonsense (NS), and splice-site (SS) pathologic variants of the gene; and deletions and insertions (producing frameshift mutations) have been shown to cause CD and ACD. When CD is detected in a clinical setting, clinicians and patients often search the Internet for information regarding pregnancy and newborn outcomes. These basic searches often describe poor outcomes, indicating that this dysplasia is nearly always lethal (90% of cases). However, upon deeper examination, this does not reflect the overall reality, especially for ACD, which is usually grouped with and often described as CD.

In this study, we present a case of ACD caused by an MS pathologic variant; the outcome is described, and we provide a review of the literature to better clarify outcomes in this disorder. In this review, we will show that the overall outcome is better in cases with ACD than in those with CD and will hopefully provide better guidance for patients and genetic counseling in the future when faced with this skeletal dysplasia in the clinical setting.

## 2 Methods/Case

We reviewed more than 1,500 abstracts, retrieved more than 500 manuscripts, and ultimately included 264 total references. This review was highly complex because at least one-third of the included publications were not identified through search engines but were located through references cited in the retrieved articles. There were numerous patient duplications in studies published prior to 1990, before *SOX9* testing, primarily involving newborns with CD. However, it was not possible to fully separate all of these within each publication due to a lack of necessary information. In contrast, cases of ACD and CD with specific genetic causes are not duplicated.

Our case involves a healthy 32-year-old G3P0020 with no major medical conditions. A twin gestation was diagnosed, with twin A being male and twin B being female. Non-invasive prenatal testing was normal. At 19 weeks’ gestation, there was a possibility of micrognathia in twin B, which was confirmed at 22 weeks’ gestation. Fetal echocardiograms were normal. As the pregnancy progressed, head measurements for twin B were increasing while long bone measurements decreased. The increase in head measurement reached significance at approximately 29 weeks’ gestation (4 weeks ahead of dates at the 99th percentile), while long bone measurements reached significance at approximately 32 weeks gestation (4 weeks behind dates at the third percentile). Mild polyhydramnios was observed in twin B starting at approximately 25 weeks gestation up to delivery, with the maximum vertical pockets ranging between 8 and 11 cm. Delivery occurred at 35 weeks’ gestation via cesarean, resulting in twin A, male, birthweight 2,440 g, and twin B, female, birthweight 2,430 g. Apgar scores for both were 7 and 9 (at 1 and 5 min).

The neonatal course was as expected for twin A, a 35-week newborn. Twin B was pink at birth but eventually required intubation due to moderate respiratory compromise in the delivery room. She was mechanically ventilated for only 7 days and was subsequently managed on CPAP and high-flow nasal cannula.

**FIGURE 1:**
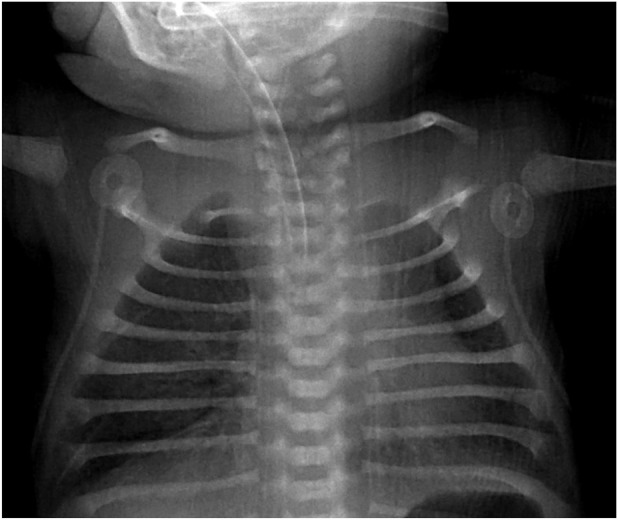
Hypoplastic scapulae.

**FIGURE 2:**
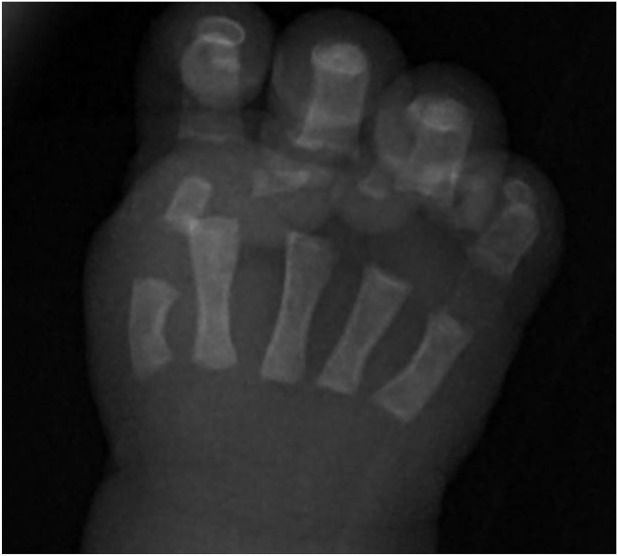
Shortened first metacarpal bone.

The skeletal survey revealed that the long bones were not bowed. Both scapulae were hypoplastic, and the first metacarpal bone was shortened (Figures 1, 2). The thoracic vertebral pedicles were not hypoplastic, but there were some abnormalities with the cervical vertebrae. The iliac wings were not vertical, and the ischiopubic bones had normal ossification. Her hips were not dislocatable, there was no equinovarus, and she had 12 pairs of ribs. Her right elbow was normal at birth but became fused at 7 months of age.

Physical findings included low-set, posteriorly rotated ears, a flat nasal bridge, and a long philtrum. There was moderate-to-severe micrognathia with a cleft of the soft palate (consistent with Pierre Robin sequence). All four extremities were noted to be shortened, less than the third percentile. Cranial and renal ultrasounds were normal, and an echocardiogram revealed only a small patent ductus arteriosus.

Postnatal genetic analysis revealed that she had a 46 XX karyotype with one MS pathologic variant in the *SOX9* gene. A glutamic acid (glutamate) amino acid replaced a lysine amino acid (c.517A>G/p. K173E), which is consistent with CD, though in this case, it was ACD because the femur and tibial bones were not bowed.

She primarily experienced positional respiratory events secondary to the micrognathia and was weaned to room air after 2 months of support. She required G-tube placement for nutritional support and was discharged home after 55 days in the NICU. Two weeks later, she underwent mandibular distraction, and 7 months after discharge, her soft palate cleft was repaired. She was sitting up at 9 months and walking independently by 19 months. At 16 months, the G-tube was removed, and she was consuming solids and liquids orally. Currently, our patient is 3 years old with no signs of scoliosis, and the only developmental delay (as evaluated by pediatric specialists) is speech-related, for which she is undergoing therapy. According to the Centers for Disease Control and Prevention pediatric growth charts, her height is at the fifth percentile, her weight is at the 40th percentile, and her head circumference is unchanged from birth and stable at the 99th percentile.

## 3 Results

Missense pathologic variants: Our case involved an MS pathologic variant that led to ACD. In our review, we identified an additional 42 cases of MS pathologic variants (Table 1). Of these (including this case), 21 were patients with ACD (Friedrich et al., 1992; Friedrich et al., 2000; Thong et al., 2000; Moog et al., 2001; Mansour et al., 2002; Bernard et al., 2003; Sock et al., 2003; Michel-Calemard et al., 2004; Wada et al., 2009; Staffler et al., 2010; Corbani et al., 2011; Matsushita et al., 2013; Gopakumar et al., 2014; Takenouchi et al., 2014; Kaissi et al., 2016; Preiksaitiene et al., 2016; von Bohlen et al., 2017; Matsumoto et al., 2018; Miller et al., 2022). There were ten 46 XY male individuals, ten 46 XX female individuals, and one 46 XY female individual with a disorder of sex development (DSD). Excluding pregnancy terminations and no newborn outcome reported, 17 (94%) of the 18 remaining cases were alive at the last reported follow-up, with 13 (75%) of them aged 2 years or older.

**TABLE 1:** Missense pathologic variants: acampomelic campomelic dysplasia, campomelic dysplasia, and other.

Case #	Disorder	Pathologic variant	Author/Year	Karyotype	Phenotypic sex	Familial vs. *De novo*	Newborn/Child outcome^a^
1	ACD	c.193C>T/p. H65Y	Mansour, 2002	46 XY	Male	Familial	Alive at the last reported follow-up (11 years)
2	ACD	c.227C>A/p. A76E	Sock, 2003	46 XY	Male	*De novo*	Alive at the last reported follow-up (6 years)
3	ACD	c.227C>A/p. A76E	Bernard, 2003	46 XY	Male	*De novo*	NR
4	ACD	c.236A>C/p. Q79P	Matsumoto, 2018	46 XY	Male	*De novo*	Alive at the last reported follow-up (26 months)
5	ACD	c.239T>G/p. V80G	Takenouchi, 2014	46 XX	Female	*De novo*	Alive at the last reported follow-up (24 months)
6	ACD	c.316A>G/p. K106E	Preiksaitiene, 2015	46 XX	Female	*De novo*	Alive at the last reported follow-up (6 months)
7	ACD	c.337A>G/p. M113V	Staffler, 2010	46 XX	Female	*De novo*	Alive at the last reported follow-up (24 months)
8	ACD	c.454C>T/p. R152W	Miller, 2022	46 XX	Female	*De novo*	Alive at the last reported follow-up (4 months)
9	ACD	c.455G>C/p. R152P	Friedrich, 2000	46 XX	Female	*De novo*	Neonatal death
10	ACD	c.493C>T/p. H165Y	Moog, 2001	46 XY	Male	*De novo*	Alive at the last reported follow-up (22 months)
11	ACD	c.495C>G/p. H165Q	Staffler, 2010	46 XY	Male	*De novo*	Alive at the last reported follow-up (8 years)
12	ACD	c.507C>G/p. H169Q	Matsushita, 2013^b^	46 XY	Male	Familial	Alive at the last reported follow-up (10 years)
13	ACD	c.507C>G/p. H169Q	Matsushita, 2013	46 XX	Female	*De novo*	Alive at the last reported follow-up (30 years)
14	ACD	c.509C>T/p. P170L	Wada, 2009	46 XY	Male	*De novo*	Alive at the last reported follow-up (11.5 years)
15	ACD	c.509C>T/p. P170L	Corbani, 2010	46 XX	Female	*De novo*	Alive at the last reported follow-up (15 years)
16	ACD	c.509C>G/p. P170R	Kaissi, 2016	46 XX	Female	*De novo*	Alive at the last reported follow-up (10 years)
17	ACD	c.517A>G/p. K173E	Thong, 2000	46 XY	Male	*De novo*	Alive at the last reported follow-up (12 months)
18	ACD	c.517A>G/p. K173E	Present case, 2026	46 XX	Female	*De novo*	Alive at the last reported follow-up (3 years)^c^
19	ACD	c.527C>T/p. P176L	Michel-calemard, 2004	46 XY DSD	Female	*De novo*	Pregnancy terminated^c^
20	ACD	c.527C>T/p. P176L	Gopakumar, 2014	46 XY	Male	*De novo*	NR^c^
21	ACD	c.185G>A^d^	Von Bohlen, 2017	46 XX	Female	*De novo*	Alive at the last reported follow-up (15 years)^c^
22	OD^e^	c.718A>C/p. T240P	Ettaki, 2025	46 XY	Male	*De novo*	Alive at the last reported follow-up (10 years)
23	DSD	c.1180C>G/p. R394G	Katoh-Fukui, 2015	46 XY DSD	Female	*De novo*	Alive at the last reported follow-up (19 years)
24	DSD	c.1309C>T/p. R437C	Katoh-Fukui, 2015	46 XY DSD	Female	Familial	Alive at the last reported follow-up (5 years)
25	CVM^e^	c.1405A>G/p.M469V	Wu, 2019	NR	NR	NR	Alive at the last reported follow-up (3 years)
26	CD	c.314A>G	Gentilin, 2010	46 XX	Female	*De novo*	Pregnancy terminated
27	CD	c.323C>T/p. P108L	Meyer, 1997	46 XY DSD	Female	NR	Infant death
28	CD	c.334T>C/p. F112L	Kwok, 1995	46 XY	Male	NR	Neonatal death
29	CD	c.335T>C/p. F112S	Goji, 1998	46 XY	Male	NR	Alive at the last reported follow-up (8 years)
30	CD	c.338T>C/p. M113T	Wada, 2009	46 XX	Female	*De novo*	Infant death
31	CD	c.356C>T/p. A119V	Kwok, 1995	46 XX	Female	NR	Neonatal death
32	CD	c.356C>T/p. A119V	Jakubiczka, 2001	46 XX	Female	*De novo*	Alive at the last reported follow-up (30 months)
33	CD	c.358C>T/p. R120C	Barone, 2014	46 XX	Female	*De novo*	Pregnancy terminated
34	CD	c.427T>C/p. W143R	Meyer, 1997	46 XY DSD	Female	NR	Infant death
35	CD	c.462C>G/p. F154L	Preiss, 2001	46 XX	Female	NR	Neonatal death
36	CD	c.472G>A/p. A158T	Preiss, 2001	46 XY DSD	Female	NR	Alive at the last reported follow-up (18 years)
37	CD	c.473C>T/p. A158V	Karaer, 2014	46 XX	Female	*De novo*	Alive at the last reported follow-up (1 month)
38	CD	c.493C>T/p. H165Y	McDowall, 1999	46 XY	Male	Familial	Alive at the last reported follow-up (10 years)
39	CD	c.494A>C/p.H165P	Tonni, 2013	46 XY	Male	*De novo*	Pregnancy terminated
40	CD	c.506A>C/p. H169P	Massardier, 2008	46 XY DSD	Female	*De novo*	Pregnancy terminated
41	CD	c.509C>G/p. P170R	Meyer, 1997	46 XY	Male	*De novo*	Neonatal death
42	CD	c.509C>T/p. P170L	Mansour, 2002	46 XX	Female	*De novo*	Alive at the last reported follow-up (21 years)
43	CD	c.1320G>A	Tonni, 2016	46 XY	Male	NR	Pregnancy terminated

^a^ Months (mos) below age 3/years (yrs) ≥ three.

^b^ Patient’s mother (case number 13) had the same defect with milder clinical/physical findings.

^c^ Twin.

^d^ This missense pathologic variant created a start codon (ATG) leading to a 62-codon open reading frame from the wild-type start codon, producing a mutant protein that reduced the expression of the normal SOX9 protein.

^e^ Physical description in these cases could represent very mild ACD.

ACD, acampomelic campomelic dysplasia; CD, campomelic dysplasia; CVM, congenital vertebral malformations; DSD, disorder of sex development; OD, odontogenesis defect; NR, not reported.

A total of 18 of the MS pathologic variants produced CD (Table 1) (Mansour et al., 2002; Wada et al., 2009; Kwok et al., 1995; Meyer et al., 1997; Goji et al., 1998; McDowall et al., 1999; Jakubiczka et al., 2001; Preiss et al., 2001; Massardier et al., 2008; Gentilin et al., 2010; Tonni et al., 2013; Barone et al., 2014; Karaer et al., 2014; Tonni et al., 2016). There were six 46 XY male individuals, eight 46 XX female individuals, and four 46 XY DSD female individuals. Excluding pregnancy terminations, 6 (46%) of the 13 remaining cases were alive at the last reported follow-up, with 5 of them aged 2 years or older. There were seven (54%) who died at birth or up to approximately 6 months of age. Finally, we also discovered four MS pathologic variants that primarily produced odontogenesis (OD), DSD, and congenital vertebral malformations (CVM) (Katoh-Fukui et al., 2015; Wu et al., 2019; Ettaki et al., 2025).

Chromosome 17 structural rearrangements: A total of 31 cases had chromosome 17 structural rearrangements (Table 2). There were 22 that produced ACD (20 translocations and 2 inversions) (Mansour et al., 2002; Kwok et al., 1995; Tommerup et al., 1993; Ninomiya et al., 1995; Ninomiya et al., 1996; Wirth et al., 1996; Savarirayan and Bankier, 1998; Pfeifer et al., 1999; Stalker and Zori, 1997; Stalker et al., 2001; Hill-Harfe et al., 2005; Velagaleti et al., 2005; Jakobsen et al., 2007; Leipoldt et al., 2007; Jakubiczka et al., 2010; Fukami et al., 2012; Fonseca et al., 2013; Khor et al., 2014; Walters-Sen et al., 2014; Morozumi et al., 2018; Takano et al., 2021; Waszyn et al., 2025). Of these cases, 18 (82%) were alive and beyond infancy at the last reported follow-up (14 of whom were aged 2 years or older).

**TABLE 2:** Chromosomal structural rearrangements involving chromosome 17 in the *SOX9* gene region.

Case #	Disorder	Chromosome 17 finding	Author/Year	Rearrangement	Newborn/Child outcome^a^
1	ACD	Translocation t. (1; 17) (q42.1; q24.3)	Leipoldt, 2007	Balanced	Alive at the last reported follow-up (5 years)
2	ACD	Translocation t. (2; 17) (q23.3; q24.3)	Jakobsen, 2007	Balanced	Alive at the last reported follow-up (15 years)
3	ACD	Translocation t. (2; 17) (p11.1; q24.2)	Khor, 2014	NR	Alive at the last reported follow-up (23 years)
4	ACD	Translocation t. (2; 17) (p15; q24.2)	Morozumi, 2018	NR	Alive at the last reported follow-up (7 years)
5	ACD	Translocation t. (4; 17) (q28.3; q24.3)	Velagaleti, 2005	Balanced	Alive at the last reported follow-up (6 years)
6	ACD	Translocation t. (4; 17) (q21.3; q23.3)	Mansour, 2002	Balanced	Alive at the last reported follow-up (11 years)
7	ACD	Translocation t. (5; 17) (q22; q23.1)	Wasyn, 2005	Balanced	Alive at the last reported follow-up (12 months)
8	ACD	Translocation t. (5; 17) (q15; q25.1)	Savarirayan, 1998	Balanced	Neonatal death
9	ACD	Translocation t. (6; 17) (q25.3; q24)	Walters-Sen, 2014	Balanced	Alive at the last reported follow-up (16 months)
10	ACD	Translocation t. (6; 17) (q14; q24)	Wirth, 1996	NR	Alive at the last reported follow-up (24 months)
11	ACD	Translocation t. (7; 17) (p13; q24)	Fonseca, 2013	Balanced	Alive at the last reported follow-up (32 years)
12	ACD	Translocation t. (7; 17) (q33; q24)	Jakubiczka, 2010	Unbalanced	Alive at the last reported follow-up (5 years)
13	ACD	Translocation t. (11; 17) (p15.4; q24.3)	Takano, 2021	Balanced	Alive at the last reported follow-up (35 years)
14	ACD	Translocation t. (12; 17) (q21.32; q24.3 or 25.1)	Ninomiya, 1995	NR	Infant death
15	ACD	Translocation t. (13; 17) (q22.1; q23.2)	Stalker, 2001	Balanced	Alive at the last reported follow-up (29 years)
16	ACD	Translocation t. (13; 17) (q22; q25.1)	Tommerup, 1993	Balanced	Alive at the last reported follow-up (28 years)
17	ACD	Translocation t. (17; 20) (q24.3; q11.2)	Fonseca, 2013	Balanced	Alive at the last reported follow-up (3 years)
18	ACD	Translocation t. (17; 22) (q25.1; p11.2)	Pfeifer, 1999	Balanced	Child death (6 years)
19	ACD	Translocation t. (Y; 17) (q11.2; q24.3)	Leipoldt, 2007	Balanced	Alive at the last reported follow-up (24 months)
20	ACD	Translocation t. (4; 7;8; 17)^b^	Velagaleti, 2005	Balanced	Neonatal death
21	ACD	Inversion (17) (q21.31; q24.3)	Fukami, 2012	​	Alive at the last reported follow-up (19 months)
22	ACD	Inversion (17) (q11.2; q24.2–25.1)	Kwok, 1995	​	Alive at the last reported follow-up (21 months)
23	CD	Translocation t. (1; 17) (q42.13; q24.3 or q25.1)	Tomerrup, 1993^c^	Balanced	Alive at the last reported follow-up (4 years)
24	CD	Translocation t. (2; 17) (q35; q23-24)	Young, 1992	Balanced	Pregnancy terminated
25	CD	Translocation t. (5; 17) (q13.3; q24.2)	Pfeifer, 1999	Balanced	Alive at the last reported follow-up (12 years)
26	CD	Translocation t. (6; 17) (p21.1; q24.3)	Antwi, 2018	Unbalanced	Alive at the last reported follow-up (2 months)
27	CD	Translocation t. (7; 17) (q32; q24.2) or (q34; q25.1)	Tomerrup, 1993^c^	Balanced	Alive at the last reported follow-up (6 years)
28	CD	Translocation t. (9; 17) (NR)	Wunderle, 1998	NR	Alive at the last reported follow-up (3 years)
29	CD	Translocation t. (10; 17) (q24; q23)	Pfeifer, 1999	Balanced	Alive at the last reported follow-up (12 months)
30	CD	Inversion (17) (q12; q25)	Maraia, 1991	​	Infant death
31	CD	Inversion (17) (q11.2; q24.3–25.1)	Mansour, 2002	​	Alive at the last reported follow-up (7 years)

^a^ Months (mos) below age 3/years (yrs) ≥ 3.

^b^ (4qter—4p15.117q25.1—17qter; 7qter—7p15.34p15.1—4pter; 8pter—8q12.17p15.3—7pter; 17pter—17p25.18q12.1—8qter).

^c^ Author states these cases were less affected (possible ACD).

ACD, acampomelic campomelic dysplasia; CD, campomelic dysplasia; NR, not reported.

Nine chromosome 17 structural rearrangements led to CD (seven translocations and two inversions) (Table 2) (Mansour et al., 2002; Tommerup et al., 1993; Maraia et al., 1991; Young et al., 1992; Antwi et al., 2018). Excluding one pregnancy termination, seven of the eight (88%) were alive at the last reported follow-up (five were aged 3 years or older). For the 27 translocations, excluding five patients with non-reporting, 20 of the 22 were described as balanced and two were reported as unbalanced (Table 2).

Deletions: A total of 27 deletion cases were identified that involved chromosome 17 in the *SOX9* gene region. These deletions ranged in size from one nucleotide to up to 2.3 mb of nucleotides, producing frameshift mutations. A total of 10 of these deletion cases produced ACD, and all 10 were alive at the last reported follow-up (2–31 years of age) (Mansour et al., 2002; Pop et al., 2004; Lecointre et al., 2009; Chen et al., 2012; Lestner et al., 2012; Bhagavath et al., 2014; Castori et al., 2016).

There were 17 deletion cases that led to CD (Mansour et al., 2002; Wada et al., 2009; Meyer et al., 1997; Gentilin et al., 2010; Ebensperger et al., 1991; Olney et al., 1999; Ninomiya et al., 2000; Smyk et al., 2007; Cost et al., 2009; Kim et al., 2011; Nelson et al., 2011; Mattos et al., 2015; Higeta et al., 2018; Kayhan et al., 2019; Liu et al., 2022). Excluding two pregnancy terminations and one case in which newborn outcome was not reported, 10 (71%) of the remaining 14 cases died at birth or up to 9 months of age. Of the four surviving CD cases, three were aged 5 years or older and one was alive at 11 months of age.

Insertions: A total of 19 insertion cases were found, with one that produced ACD in a pregnancy that was ultimately terminated (Bergeron et al., 2009). These insertions were all single-nucleotide insertions, except for three that had insertions of 4 base pairs, 5 base pairs, and 16 base pairs.

Of the 18 insertions that led to CD, 5 pregnancies were terminated, and for two patients, newborn outcome was not reported (Wada et al., 2009; Kwok et al., 1995; Gentilin et al., 2010; Camer et al., 1996; Giordano et al., 2001; Watiker et al., 2005; Hsiao et al., 2006; Okamoto et al., 2010; Carvajal et al., 2016). Of the remaining 11 cases, 8 (73%) died on the day of birth or up to 4 months of age. One died at 5 years of age, and two others were alive at the last reported follow-up (aged 5 years).

Nonsense pathologic variants: We identified 26 NS pathologic variants, and all of these resulted in CD (Table 3) (Wada et al., 2009; Meyer et al., 1997; Massardier et al., 2008; Mattos et al., 2015; Watiker et al., 2005; Foster et al., 1994; Wagner et al., 1994; Hageman et al., 1998; Pop et al., 2005; Shotelersuk et al., 2006; Stoeva et al., 2011; Amano et al., 2019; Csukasi et al., 2019; Zhen et al., 2019; Calvache et al., 2022; Qiao et al., 2022). Excluding pregnancy terminations and cases with no reported newborn outcome, of the remaining 18 cases, 13 (72%) died, 11 on the day of birth or up to 7 months of age. Of the five who were alive at the last reported follow-up, four were aged 2–12 years.

**TABLE 3:** Nonsense pathologic variants: campomelic dysplasia^a^.

Case #	Pathologic variant	Author/Year	Familial vs. de novo	Newborn/Child outcome^b^
1	p. E28X	Massardier, 2008	*De novo*	Pregnancy terminated
2	p. S41X	Mattos, 2015	NR	Neonatal death
3	p. W86X	Meyer, 1997	*De novo*	Neonatal death
4	p. W115X	Calvache, 2022	*De novo*	Alive at the last reported follow-up (4 years)
5	p. Q117X	Meyer, 1997	*De novo*	Alive at the last reported follow-up (12 years)
6	p. E148X	Wagner, 1994	Familial^c^	Neonatal death
7	p. E148X	Wada, 2009	*De novo*	Infant death
8	p. E150X	Shotelersuk, 2006	NR	Infant death
9	p. K151X	Mattos, 2015	NR	Neonatal death
10	p. Q175X	Amano, 2019	NR	Alive at the last reported follow-up (27 months)
11	p. ?195X	Foster, 1994	*De novo*	NR
12	p. Q232X	Mattos, 2015	NR	Neonatal death
13	p. Y297X	Watiker, 2005	NR	Neonatal death
14	p. Y319X	Csukasi, 2019	NR	Pregnancy terminated
15	p. W335X	Zhen, 2019	*De novo*	Pregnancy terminated
16	p. Q375X	Meyer, 1997	*De novo*	Infant death
17	p. Q391X	Csukasi, 2019	NR	Pregnancy terminated
18	p. R394X	Csukasi, 2019	NR	Pregnancy terminated
19	p. E400X	Meyer, 1997	NR	Infant death
20	p. Q401X	Stoeva, 2011	*De novo*	Alive at the last reported follow-up (4 months)
21	p. Q412X	Csukasi, 2019	NR	Pregnancy terminated
22	p. Q417X	Qiao, 2023	*De novo*	Pregnancy terminated
23	p. Y440X	Wagner, 1994	*De novo*	Child death (4 years)
24	p. Y440X	Meyer, 1997	*De novo*	Alive at the last reported follow-up (11 years)
25	p. Y440X	Hageman, 1998	*De novo*	Child death (4 years)
26	p. Y440X	Pop, 2005^d^	*De novo*	Infant death

^a^ Every case with a NS mutation had CD.

^b^ Months (mos) below age 3/years (yrs) ≥ 3.

^c^ Maternal mosaic (lymphocytes) with no physical abnormalities.

^d^ This case was homozygous for p. Y440X and *de novo*. This reference also states they have seven more cases in their database. Of these, one had no outcome data, two pregnancies were terminated, one was neonatal death, one was infant death, and two were alive at the last reported follow-up (24 months and 11 years). However, no other data on these cases were supplied.

NR, not reported.

Splice-site pathologic variants: There were four SS pathologic variants, with one that did not provide newborn data (Kwok et al., 1995; Mattos et al., 2015; Wagner et al., 1994). The remaining three were all CD cases, and all three died as neonates.

Negative *SOX9* gene testing: We identified 11 cases where the *SOX9* gene testing was reported as negative. However, it does not appear that full genetic testing upstream of *SOX9* was performed. Of these, five had ACD, and excluding one where newborn outcome was not supplied, the other four were still alive (three “survived childhood,” and one was alive at 6 years) (Wada et al., 2009; Meyer et al., 1997; Herman et al., 2012).

The other six had CD, with one case where the newborn outcome was not reported (Kwok et al., 1995; Nelson et al., 2011; Mattos et al., 2015; Wagner et al., 1994). Of the remaining five cases, three died at birth or during the neonatal period, one died at 10 months of age, and one was alive at the last reported follow-up (age 6 years).

Prior to or without genetic testing: There were 26 cases of ACD derived from studies conducted prior to genetic screening for *SOX9* abnormalities or in which *SOX9* testing was not performed (Lindgren and Ringertz, 1980; Friedrich et al., 1992; Kwok et al., 1995; Stalker and Zori, 1997; Stalker et al., 2001; Bricarelli et al., 1981; Macpherson et al., 1989; Decsi and Botykai, 1992; Lynch et al., 1993; Rodríguez, 1993; Glass and Rosenbaum, 1997; Offiah et al., 2002; Ozkilic et al., 2002; Unger, 2005; Pasupathy et al., 2016; Li et al., 2024). Of these cases, 10 died at delivery or up to 1 year of age, and one died at 4 years of age. Fourteen were alive at 2 years of age or older, and one was alive at less than 1 year.

There were 164 cases of CD (excluding pregnancy terminations and cases with no reported newborn outcome) derived from studies conducted prior to genetic screening for *SOX9* or in which *SOX9* testing was not performed (Supplemental Appendix 1). Of these cases, 143 (87%) died at delivery or up to 1 year of age. Twenty-one (13%) were alive at 1 year of age or older, of whom 16 were aged more than 2 years.

Combined data: In summary, through our extensive literature search, we identified 85 non-duplicated cases of ACD. Of these, 54 had complete *SOX9* gene testing. Excluding pregnancy terminations and cases with no reported newborn outcomes, 45 (90%) of the 50 remaining cases were alive at the last reported follow-up, with 86% aged over 1 year and 74% aged 2 years or older.

We found more than 250 cases of CD, but an exact number beyond this is not possible due to an inability to clearly rule out duplicated patients. However, of these, 92 non-duplicated CD patients underwent complete *SOX9* gene testing. Excluding pregnancy terminations and cases with no reported newborn outcomes, only 20 (30%) of the 67 remaining cases were alive at more than 1 year of age. Notably, regarding CD cases with *SOX9* gene assessment, survival beyond 1 year of age was approximately 25% or less with deletions, insertions, NS-pathologic variants, and SS-pathologic variants. It was approximately 40% with MS pathologic variants but 75% with chromosome 17 structural rearrangements.

## 4 Discussion

As stated in the Introduction, CD and ACD were found to be caused by genetic abnormalities of the *SOX9* gene located on the long arm of chromosome 17. It was later determined that CD and ACD are autosomal dominant haploinsufficiency genetic disorders, meaning that one functional gene copy is insufficient to produce normal protein function. The *SOX9* gene codes for a 509-amino acid protein. It contains a high-mobility-group (HMG) domain, which is a DNA-binding motif that allows *SOX9* to recognize and bind to specific DNA sequences. This HMG domain is approximately 79 codons in length, spanning positions approximately 101 to 180. The 39– 40 codons preceding the HMG domain toward the N-terminal end constitute the dimerization (DIM) domain. In addition, there are two transactivation domains: a middle domain (TAM) and a C-terminal domain (TAC). Between these two transactivation domains is a region rich in proline, glutamine, and alanine (PQA). In analyzing the 21 MS pathologic variant ACD cases, 6 occurred in the DIM domain and 15 occurred in the HMG domain. In contrast, among the 18 MS-pathologic variant CD cases, all occurred in the HMG domain except one, which was located in the TAC domain. Finally, it has been shown that the DNA upstream (up to approximately 1.5 mb) of the *SOX9* gene contains enhancers and promoters that regulate the *SOX9* gene itself. This is an important finding because complete genetic screening for this dysplasia requires both the *SOX9* gene and upstream regions.

The SOX9 protein is important for promoting the differentiation of precursor cells into chondrocytes during cartilage formation in the limbs and other skeletal areas, including the scapulae, vertebrae, iliac bones, ischiopubic bones, jaw, and other skeletal structures. This gene is also involved in the formation of a normal palate, dentition, male sex determination (in the presence of a Y chromosome), and the development of various other organs.

Skeletal findings in CD must include bowed or bent femur or tibial bones or both (a dimple may occur in the skin over the tibial site of the bending). Other skeletal findings can involve hypoplastic scapulae, poorly mineralized thoracic or cervical vertebrae, poor ossification of the ischiopubic bones, vertical iliac wings, short first metacarpal bones, dislocatable hips, equinovarus, and 11 pairs of ribs. Facial findings can include midface hypoplasia, a flat nasal bridge, hypertelorism, and a long philtrum. CD can also be associated with micrognathia, glossoptosis, and cleft palate (Pierre Robin sequence—PRS—OMIM261800). The cleft palate is usually the soft palate or may include the very distal hard palate. Finally, there is also a potential for having a 46 XY female DSD. Newborns/infants usually succumb to respiratory distress caused by tracheomalacia.

With the advancements in the field of genetics, the differential for CD (bent lower-extremity long bones) from a skeletal dysplasia standpoint has become large, as observed in Table 4. Many of these disorders are associated with additional anomalies that can be detected prenatally to help narrow the diagnosis. However, another important finding is that the only difference between CD and ACD is a bent femur or tibial bones or both. Therefore, when describing these cases, the skeletal findings of the bent femur or tibial bones or both should be mentioned first. If the bones are straight, the next most important skeletal finding to analyze is the scapulae. If they are hypoplastic, the differential is small (Table 4).

**TABLE 4:** Differential for prenatal identified bent femur and/or tibial bones and differential for hypoplastic scapulae (listed alphabetically).

Bent femur or tibial bones or both
Achondroplasia
Antley–Bixler syndrome
Campomelic dysplasia
Cumming syndrome
Diastrophic dysplasia
Hypophosphatasia
Kniest dysplasia
Kyphomelic dysplasia
Langer mesomelic dysplasia
Larson syndrome
Osteogenesis imperfecta types 2 and 3
Schwartz–Jampel syndrome
Spondyloepiphyseal dysplasia congenita
Stuve–Wiedemann syndrome
Thanatophoric dysplasia
Weismann-Netter–Stuhl syndrome

Hypoplastic scapulae
Acampomelic campomelic dysplasia
Antley–Bixler syndrome
Campomelic dysplasia
Cleidocranial dysplasia

Because these chromosomal translocations or inversions, pathologic variants, deletions, and insertions are sporadic, predicting or preventing this disorder is not possible. In addition, prenatal diagnosis of CD is likely to occur if prenatal anatomy ultrasound scans are performed because of the bent femur or tibial bones or both. However, an exact prenatal diagnosis of ACD prior to birth is harder. Prenatal ultrasound cannot reliably determine the ossification status of bones, and many findings are non-specific. Micrognathia and equinovarus can be identified prior to delivery, but these findings are not specific to CD or ACD. In most clinical scenarios, they would be considered isolated findings or, in the case of micrognathia, attributed to Pierre Robin disorder. Prenatal diagnosis of a soft-palate cleft is also difficult, especially if there is no abnormality of the hard palate, as observed in this case. Nearly all other clinical and radiographic findings are subtle and are not specific to CD or ACD in the prenatal setting. In a clinical setting, if several subtle abnormalities are observed prenatally, full genetic testing might be offered.

As stated above, if whole-genome testing is not performed, the genetic assessment of the *SOX9* gene should include evaluation of the upstream region of *SOX9*. This is necessary because numerous CD/ACD cases have abnormalities in these regions (as suggested by our 11 patients who most likely did not undergo complete genetic analysis). In addition, in cases that have 46 XY female DSD, an evaluation for gonadectomy is needed due to an increased risk for gonadoblastoma or other germ-cell tumors.

One study limitation is that numerous publications used cases that were previously reported, and it was extremely difficult to fully clarify potential patient duplications in this process. However, this only occurred in the CD and ACD cases that did not have *SOX9* genetic testing. In addition, since one-third of the cases we identified were obtained from the references cited in the retrieved studies rather than directly from Internet searches, a small number of CD or ACD cases may still be missing. We discovered that many manuscripts do not clearly specify whether the femurs or tibias were bowed, yet label the cases as CD. However, bent femur or tibial bones are the only difference between CD and ACD and should be the first clinical/radiographic report described. Clarifying this distinction in future publications will make it easier for readers to differentiate CD from ACD. This is of prime importance because they have markedly different outcomes. Many studies also report their cases as being CD, but in the body of the manuscript, they state that the long bones were not bowed. In these instances, we relabeled them as ACD.

Our case is the second reported to have the c.517A>G/p. K173E missense pathologic variant. In the prior case described by Thong et al. (2000), the newborn experienced early respiratory issues that improved over time and had similar physical and radiographic findings. However, he had 11 pairs of ribs, whereas our case had the normal 12 pairs. In that study, newborn follow-up was limited to 1 year, at which time “he was doing well.” More than 80% of ACD cases caused by MS pathologic variants survive beyond infancy.

Chromosome 17 structural rearrangements result in neonatal anomalies depending on the DNA breakpoint of the translocation or inversion. Most of these breakpoints occur upstream from the *SOX9* gene. These breakpoints can produce SOX9 proteins that are truncated or elongated, leading to decreased protein function. However, 82% of ACD cases and 75% of CD cases survived infancy. Additionally, 100% of the 10 ACD cases caused by deletions were alive at ages of 2 years or older.

It is not unexpected that NS and SS pathologic variants all produced CD cases. This is likely caused by the poorly functioning truncated or abnormal proteins produced during translation that occur with these pathologic variants. None of the SS mutation cases survived the neonatal period. Survival beyond 1 year of age with NS pathologic variants was only 22%.

Our review clearly shows that newborn outcomes in ACD are better compared with overall outcomes in CD cases, with the exception of chromosome 17 structural rearrangements. Nearly 85% of the MS pathologic variants associated with ACD (as observed in our case) survived beyond infancy, whereas survival beyond 1 year of age for cases with chromosome 17 structural rearrangements was approximately 80%; for deletions, survival was 100%.

When faced with bent femur, tibial bones, or both in a clinical setting, we recommend that the providers refer to Table 4 and consider the differential diagnosis. Some of these can be confirmed with other ultrasound findings. However, if the diagnosis cannot be firmly made, then whole-genome sequencing can be offered and performed on amniotic fluid. Several of the dysplasia types reported in Table 4 are caused by various gene mutations that would be identified through this process. Once a diagnosis of CD is made, the specific *SOX9* gene alteration should be assessed. Following this, thorough and informed counseling should be provided, including descriptions of the typically poor outcomes associated with each type of gene anomaly, except in the case of chromosome 17 structural rearrangements. For ACD, prenatal diagnosis is not likely because of the subtle, non-specific findings. However, if the diagnosis is made prenatally, then the same process would occur regarding patient counseling, though the outcome data are more reassuring.

## 5 Conclusion

In conclusion, this extensive review clearly shows that approximately 9 out of 10 cases of ACD with a genetic diagnosis over the past 30 years survive beyond 1 year of age, most of whom are over 2 years. In stark contrast, only approximately 3 out of 10 CD cases (with a genetic diagnosis in the last 30 years) survive beyond 1 year of life. However, this number is skewed because survival beyond infancy has not been reported with SS pathologic variants; it is only approximately 2 in 10 for deletions, insertions, and NS pathologic variants, approximately 4 in 10 for MS pathologic variants, and nearly 8 in 10 for chromosome 17 structural rearrangements. These findings are of critical importance for patient counseling and perinatal care planning. As more cases are published over time, it would be beneficial to include them in these tables to maintain an up-to-date, ongoing database for this rare skeletal dysplasia.

## Data Availability

The data presented in the study are deposited in the OSF repository, accession number 6mrt7: https://osf.io/6mrt7/overview.
