# Supplementary material for: SOX9 gene anomalies and campomelic / acampomelic campomelic dysplasia: case report and literature review
Source: Front Genet. 2026 Mar 10;17:1755075. doi: 10.3389/fgene.2026.1755075 (PMC13010059; doi:10.3389/fgene.2026.1755075)
Supplement: Supplementary file 1 [file DataSheet1.docx]

***Supplementary Material***

1. **Campomelic Dysplasia References**
2. Gaenslen FJ. Congenital Defects of the Tibia and Fibula. JBJS. 1915;s2-12(3):453-81.
3. Snure H. Intra-Uterine Fracture: Case Report and Review of Roentgenologic Findings. Radiology. 1929;13(4):362-5.
4. 115. Middleton DS. Studies on Prenatal Lesions of Striated Muscle as a Cause of Congenital Deformity: I.-Congenital Tibial Kyphosis. II.-Congenital High Shoulder. III.-Myodystrophia Fœtalis Deformans. Edinb Med J. 1934;41(7):401-42.
5. 116. Browne D. Congenital Deformities of Mechanical Origin: (Section for the Study of Disease in Children). Proc R Soc Med. 1936;29(11):1409-31.
6. Freund E. Congenital defects of femur, fibula and tibia. Archives of Surgery. 1936;33(3):349-91.
7. Chapple CC, Davidson DT. A study of the relationship between fetal position and certain congenital deformities. The Journal of Pediatrics. 1941;18(4):483-93.
8. Williams ER. Two congenital deformities of the tibia: Congenital angulation and congenital pseudarthrosis. BR J Radiol. 1943;16:371-6.
9. Caffey J. Prenatal bowing and thickening of tubular bones, with multiple cutaneous dimples in arms and legs; a congenital syndrome of mechanical origin. Am J Dis Child (1911). 1947;74(5):543-62.
10. Dawson GR, Jr. Intra-uterine fractures of the tibia and fibula; report of a case with correction by osteotomy and plating. J Bone Joint Surg Am. 1949;31a(2):406-8.
11. Heyman CH, Herndon CH. Congenital posterior angulation of the tibia. J Bone Joint Surg Am. 1949;31a(3):571-80.
12. Krida A. Congenital posterior angulation of the tibia; a clinical entity unrelated to congenital pseudarthrosis. Am J Surg. 1951;82(1):98-102.
13. Miller BF. Congenital posterior bowing of the tibia with talipes calcaneovalgus. J Bone Joint Surg Br. 1951;33-b(1):50-5.
14. Bound JP, Finlay HV, Rose FC. Congenital anterior angulation of the tibia. Arch Dis Child. 1952;27(132):179-84.
15. Hertig AT. <i>Pathology of the Fetus and the Newborn</i>. Edith L. Potter. Chicago: Year Book Pub., 1952. 578 pp. and 601 figs. $19.00. Science. 1952;115(2996):612-.
16. Conway TJ. Prenatal bowing and angulation of long bones; a description of its occurrence in a brother and sister. AMA J Dis Child. 1958;95(3):305-8.
17. Bain AD, Barrett HS. Congenital bowing of the long bones: report of a case. Arch Dis Child. 1959;34(178):516-24.
18. Dunn AW, Aponte GE. Congenital Bowing of the Tibia and Femur: Case Report with Autopsy Findings. The Journal of Bone & Joint Surgery. 1962;44(4):737-40.
19. Rath F, Thalhammer O. [Prenatal bowing of the long tubular bones]. Z Kinderheilkd. 1967;98(1):63-74.
20. Engel W, Reinwein H, Bombel D, Ritter H, Wolf U. [Multiple abnormalities in a girl with a 46, XY,17q+ karyotype]. Humangenetik. 1968;6(4):311-25.
21. Schudel P. [Morphology of congenital bowing of highs and lower legs]. Helv Paediatr Acta. 1968;23(6):659-67.
22. Blessinger GM. Syndrome of multiple osseous deformities. Lancet. 1970;2(7680):982.
23. Gardner LI, Assemany SR, Neu RL. 46, XY female: anti-androgenic effect of oral contraceptive? Lancet. 1970;2(7674):667-8.
24. Bailey RR. Congenital bowing of the long bones. Lancet. 1971;2(7716):157-8.
25. Bain AD, Barrett HS. Congenital bowing of the long bones. Lancet. 1971;1(7711):1244.
26. Gardner LI, Assemany SR, Neu RL. Syndrome of multiple osseous defects with pretibial dimples. Lancet. 1971;2(7715):98.
27. Stüve A, Wiedemann HR. Congenital bowing of the long bones in two sisters. Lancet. 1971;2(7722):495.
28. Hoefnagel D, Wurster D, Carey D, Harris GJ, Pilliod J. Camptomelic dwarfism. Lancet. 1972;1(7759):1068.
29. Kucera J. Syndrome of multiple osseous defects. Lancet. 1972;1(7744):260-1.
30. Ladreyt JP. [Camptomelic dwarfism]. Pediatrie. 1972;27(5):567-8.
31. Lee FA, Isaacs H, Jr., Strauss J. The "campomelic" syndrome. Short life-span dwarfism with respiratory distress, hypotonia, peculiar facies, and multiple skeletal and cartilaginous deformities. Am J Dis Child. 1972;124(4):485-96.
32. Stuart Houston C, Awen CF, Kent HP. Fatal neonatal dwarfism. J Can Assoc Radiol. 1972;23(1):45-61.
33. Cremin BJ, Orsmond G, Beighton P. Autosomal recessive inheritance in camptomelic dwarfism. Lancet. 1973;1(7801):488-9.
34. Poznanski AK, Holt JF. Campomelic dwarfism. Semin Roentgenol. 1973;8.
35. Rupprecht E, Manitz U. [Disease picture of congenital bowing of long bones]. Helv Paediatr Acta. 1973;28(5):467-76.
36. Schmickel RD, Heidelberger KP, Poznanski AK. The campomelique syndrome. J Pediatr. 1973;82(2):299-302.
37. Szalay GC. The "campomelic" syndrome. Am J Dis Child. 1973;126(1):128.
38. Thurmon TF, DeFraites EB, Anderson EE. Familial camptomelic dwarfism. J Pediatr. 1973;83(5):841-3.
39. Baggio P. Sindrome campomelica famigliare. Acta Paediatr Lat. 1974;27.
40. Curran JP, Sigmon BA, Opitz JM. Lethal forms of chondrodysplastic dwarfism. Pediatrics. 1974;53(1):76-85.
41. Kucera J, Hodr R. [Etiology of campomelic syndrome]. Cesk Pediatr. 1974;29(3):156-9.
42. Mahloudji M, Zarrabi M, Emami-Ahari Z. Prenatal bowing of long bones in two sibs. Birth Defects Orig Artic Ser. 1974;10(5):121-4.
43. Opitz JM, Feingold M, Bull MJ, Spranger JW. The campomelic syndrome--comments. Birth Defects Orig Artic Ser. 1974;10(9):97-9.
44. Storer J, Grossman H. The campomelic syndrome. Congenital bowing of limbs and other skeletal and extraskeletal anomalies. Radiology. 1974;111(3):673-81.
45. Szalay GC. Letter: The camptomelic syndrome. J Pediatr. 1974;84(5):780.
46. Thurmon TF, Kityakara A. Camptomelic dwarfism. Birth Defects Orig Artic Ser. 1974;10(9):89-95.
47. Walbaum R, Scharfman W, Houcke M, Peuzin F. Le nanisme campomélique. Pediatrie. 1974;29.
48. Allain D, Lambert A. Campomelic syndrome (with reference to a case). Medecine Infantile. 1975;82(1):65-71.
49. Becker MH, Finegold M, Genieser NB, Darling D, Feingold M. Campomelic dwarfism. Birth Defects Orig Artic Ser. 1975;11(6):113-8.
50. Centa A, Pezzuto C. Incurvamento congenito delle ossa lunghe degli arti. Radiol Pratica. 1975;15.
51. Eliacher E, Baux S, Maroteaux P, Tassy R, Perraudin ML. Incurvation congenitale des os longs: Un nouveau cas. Ann Pediatr. 1975;22:61-4.
52. Polidori G, Mastroiacovo P, Cataldi L, Segni G. Picture of the month. Campomelic syndrome. Am J Dis Child. 1975;129(5):615-6.
53. Rogers JG, Cranley RE, Dorst JP, Levin LS, Williams BR. A variant of campomelia. Birth Defects Orig Artic Ser. 1975;11(6):119-25.
54. Rolland M, Dupic Y, Bergès JD, Régnier C. Le syndrome camptomélique: à propos d'une observation. Pediatrie. 1975;30:860-1.
55. Bentivoglio M, Di Trapani G, Mastroiacovo PP, Macchi G. [Brain malformations in a case of camptomelic syndrome]. Acta Neurol (Napoli). 1976;31(1):51-5.
56. Berndt KJ, Seiler R, Pfeiffer RA. [The campomelic syndrome (author's transl)]. Monatsschr Kinderheilkd (1902). 1976;124(2):93-5.
57. Camera G, Centa A. [Campomelic dwarfism. Re-examination of our study]. Minerva Pediatr. 1976;28(28):1727-30.
58. Khajavi A, Lachman R, Rimoin D, Schimke RN, Dorst J, Handmaker S, et al. Heterogeneity in the campomelic syndromes. Long-and short-bone varieties. Radiology. 1976;120(3):641-7.
59. Shafai T. Letter: Camptomelic syndrome in siblings. J Pediatr. 1976;89(3):512-3.
60. Weiner DS, Benfield G, Robinson H. Camptomelic dwarfism. Report of a case and review of the salient features. Clin Orthop Relat Res. 1976(116):29-31.
61. Hovmöller ML, Osuna A, Eklöf O, Fredga K, Hjerpe A, Linsten J, et al. Camptomelic dwarfism. A genetically determined mesenchymal disorder combined with sex reversal. Hereditas. 1977;86(1):51-62.
62. Kozlowski K, Masel J, Morris L, Ryan J, Collins E, Van Vliet P, et al. Neonatal death dwarfism. Australas Radiol. 1977;21(2):164-83.
63. Mastroiacovo P, Sagui L, Seganti G, Polidori G. La sindrome camptomelica: Considerazioni su due casi. Minerva Pediatr. 1977;29.
64. Ameri MR, Alebouyeh M, Amirfeyz M, Ziai M, Rafii MR, Gandjour A. [Campomelic syndrome (author's transl)]. Monatsschr Kinderheilkd (1902). 1978;126(11):687-9.
65. Fasanelli L, Fasanelli S. The campomelic syndrome. (A case report). Rays. 1978;3.
66. Gruhn JG, Gorlin RJ, Langer LO. Dyssegmental dwarfism. A lethal anisospondylic camptomicromelic dwarfism. Am J Dis Child. 1978;132(4):382-6.
67. Hoefnagel D, Wuster-Hill DH, Dupree WB, Benirschke K, Fuld GL. Camptomelic dwarfism associated with XY-gonadal dysgenesis and chromosome anomalies. Clin Genet. 1978;13(6):489-99.
68. Kozlowski K, Bützler HO, Galatius-Jensen F, Tulloch A. Syndromes of congenital bowing of the long bones. Pediatr Radiol. 1978;7(1):40-8.
69. Segre A, Beluffi G, Peretti G. Camptomelic syndrome. A rare type of congenital dwarfism associated with skeletal and other abnormalities. Ital J Orthop Traumatol. 1978;4(2):237-52.
70. Formantici F. [Camptomelic dwarfism. Apropos of a new observation]. Minerva Pediatr. 1979;31(8):649-56.
71. Krous HF, Turbeville DF, Altshuler GP. Campomelic syndrome--possible role of intrauterine viral infection. Teratology. 1979;19(1):9-14.
72. Schimke RN. XY sex-reversed campomelia- possibly an X-linked disorder? Clin Genet. 1979;16(1):62-3.
73. Tokita N, Chandra-Sekhar HK, Daly JF, Becker MH, Aleksic S. The Campomelic syndrome. Temporal bone histopathologic features and otolaryngologic manifestations. Arch Otolaryngol. 1979;105(8):449-54.
74. Austin GE, Gold RH, Mirra JM, Perry S, Moedjono S. Long-limbed campomelic dwarfism. A radiologic and pathologic study. Am J Dis Child. 1980;134(11):1035-42.
75. Fontaine G, Walbaum R, Farriaux JP, Tilmont P, Peuzin F, Delecour M. [Genetic counseling in campomelic dysplasia (apropos of 2 cases)]. J Genet Hum. 1980;28(3):267-79.
76. Fraccaro M, Zuffardi O, Baggio P, Console V, Valagussa E, editors. Campomelic dysplasia and sex reversal. Genetik in der Pädiatrie II Symposium, Mainz 1979; 1980: Thieme Stuttgart.
77. Hall BD, Spranger JW. Campomelic dysplasia. Further elucidation of a distinct entity. Am J Dis Child. 1980;134(3):285-9.
78. Mellows HJ, Pryse-Davies J, Bennett MJ, Carter CO. The camptomelic syndrome in two female siblings. Clin Genet. 1980;18(2):137-41.
79. Moedjono SJ, Crandall BF, Sparkes RS, Feldman GM, Austin GE, Perry S. The campomelic syndrome in a singleton and monozygotic twins. Clin Genet. 1980;18(6):397-401.
80. Paky F, Stögmann W, Holzleitner P. [Campomelic syndrome]. Padiatr Padol. 1980;15(3):245-51.
81. Pauli RM, Pagon RA. Abnormalities of sexual differentiation in campomelic dwarfs. Clin Genet. 1980;18(3):223-5.
82. Pavone L, Grasso S, Mazzone D, Sciacca F. Camptomelic dwarfism associated with camptodactyly in a new-born infant from consanguineous parents. Acta Paediatr Belg. 1980;33(2):129-32.
83. Bethem D, Winter RB, Lutter L, Moe JH, Bradford DS, Lonstein JE, et al. Spinal disorders of dwarfism. Review of the literature and report of eighty cases. J Bone Joint Surg Am. 1981;63(9):1412-25.
84. Fasani G, Torresani P, Bontardelli G. Sindrome camptomelica. Boll Soc Med Chir Cremona. 1981.
85. Filly RA, Golbus MS, Carey JC, Hall JG. Short-limbed dwarfism: ultrasonographic diagnosis by mensuration of fetal femoral length. Radiology. 1981;138(3):653-6.
86. Fryns JP, van den Berghe K, van Assche A, van den Berghe H. Prenatal diagnosis of campomelic dwarfism. Clin Genet. 1981;19(3):199-201.
87. Lenz W. [Campomelia (author's transl)]. Klin Padiatr. 1981;193(1):10-2.
88. Puck SM, Haseltine FP, Francke U. Absence of H-Y antigen in an XY female with campomelic dysplasia. Hum Genet. 1981;57(1):23-7.
89. Shah KN, Patel ZM, Desai AP, Kulkarni MV, Ambani LM. Campomelic syndrome in phenotypic females with 46,XY chromosomes: evidence of genetic heterogeneity. Clin Pediatr (Phila). 1981;20(3):214-6.
90. Trompeter RS, Shrubb V, Heaton JM, Berry AC. XY Sex-reversed campomelia. Eur J Pediatr. 1981;137(1):59-61.
91. Beluffi G, Fraccaro M. Genetical and clinical aspects of campomelic dysplasia. Prog Clin Biol Res. 1982;104:53-68.
92. Noyal P, Vermeulin G, Hibon D, Meck JM. Campomelic dwarfism. One case with a 4 year survival. Archives Francaises de Pediatrie. 1982;39(8):621-4.
93. Roth SI, Jimenez JF, Husted S, Seibert JJ, Haynes DW. The histopathology of camptomelia (bent limbs). A dyschondrogenesis. Clin Orthop Relat Res. 1982(167):152-9.
94. Bharucha BA, Savliwala AS, Koppikar GV, Kumta NB. Camptomelic dwarfism syndrome (a case report). J Postgrad Med. 1983;29(4):268-70.
95. Houston CS, Opitz JM, Spranger JW, Macpherson RI, Reed MH, Gilbert EF, et al. The campomelic syndrome: review, report of 17 cases, and follow-up on the currently 17-year-old boy first reported by Maroteaux et al in 1971. Am J Med Genet. 1983;15(1):3-28.
96. Ray S, Bowen JR. Orthopaedic problems associated with survival in campomelic dysplasia. Clin Orthop Relat Res. 1984(185):77-82.
97. Cooke CT, Mulcahy MT, Cullity GJ, Watson M, Srague P. Campomelic dysplasia with sex reversal: morphological and cytogenetic studies of a case. Pathology. 1985;17(3):526-9.
98. Lazjuk GI, Shved IA, Cherstvoy ED, Feshchenko SP. Campomelic syndrome: concepts of the bowing and shortening in the lower limbs. Teratology. 1987;35(1):1-8.
99. McAlister WH, Applewhite T, Taysi K, Gilula LA. Roentgen rounds #90. Newborn with club feet and dislocatable hips, knees, and elbows. Campomelic dysplasia. Orthop Rev. 1987;16(9):687-90.
100. Coscia MF, Bassett GS, Bowen JR, Ogilvie JW, Winter RB, Simonton SC. Spinal abnormalities in camptomelic dysplasia. J Pediatr Orthop. 1989;9(1):6-14.
101. Gillerot Y, Vanheck CA, Foulon M, Podevain A, Koulischer L. Campomelic syndrome: manifestations in a 20 week fetus and case history of a 5 year old child. Am J Med Genet. 1989;34(4):589-92.
102. Jäger RJ, Anvret M, Hall K, Scherer G. A human XY female with a frame shift mutation in the candidate testis-determining gene SRY. Nature. 1990;348(6300):452-4.
103. Argaman Z, Hammerman CA, Kaplan M, Schimmel M, Rabinovich R, Tunnessen WW, Jr. Picture of the month. Campomelic dysplasia. Am J Dis Child. 1993;147(2):205-6.
104. Normann EK, Pedersen JC, Stiris G, van der Hagen CB. Campomelic dysplasia--an underdiagnosed condition? Eur J Pediatr. 1993;152(4):331-3.
105. Hong JR, Barber M, Scott CI, Guttenberg M, Wolfson PJ. 3-Year-old phenotypic female with campomelic dysplasia and bilateral gonadoblastoma. J Pediatr Surg. 1995;30(12):1735-7.
106. Mansour S, Hall CM, Pembrey ME, Young ID. A clinical and genetic study of campomelic dysplasia. J Med Genet. 1995;32(6):415-20.
107. Schmitt-Ney M, Thiele H, Kaltwasser P, Bardoni B, Cisternino M, Scherer G. Two novel SRY missense mutations reducing DNA binding identified in XY females and their mosaic fathers. Am J Hum Genet. 1995;56(4):862-9.
108. Foster JW. Mutations in SOX9 cause both autosomal sex reversal and campomelic dysplasia. Acta Paediatr Jpn. 1996;38(4):405-11.
109. Schafer AJ, Foster JW, Kwok C, Weller PA, Guioli S, Goodfellow PN. Campomelic dysplasia with XY sex reversal: diverse phenotypes resulting from mutations in a single gene. Ann N Y Acad Sci. 1996;785:137-49.
110. Südbeck P, Schmitz ML, Baeuerle PA, Scherer G. Sex reversal by loss of the C-terminal transactivation domain of human SOX9. Nat Genet. 1996;13(2):230-2.
111. Thomas S, Winter RB, Lonstein JE. The treatment of progressive kyphoscoliosis in camptomelic dysplasia. Spine (Phila Pa 1976). 1997;22(12):1330-7.
112. Kehagias D, Pafiti A, Kalabokis D, Botsis D, Vlahos L. Campomelic dysplasia in a twin pregnancy. A case report. Clin Exp Obstet Gynecol. 1998;25(4):157-8.
113. Wunderle VM, Critcher R, Hastie N, Goodfellow PN, Schedl A. Deletion of long-range regulatory elements upstream of SOX9 causes campomelic dysplasia. Proc Natl Acad Sci U S A. 1998;95(18):10649-54.
114. Huang B, Wang S, Ning Y, Lamb AN, Bartley J. Autosomal XX sex reversal caused by duplication of SOX9. Am J Med Genet. 1999;87(4):349-53.
115. Tongsong T, Wanapirak C, Pongsatha S. Prenatal diagnosis of campomelic dysplasia. Ultrasound Obstet Gynecol. 2000;15(5):428-30.
116. Holder-Espinasse M, Abadie V, Cormier-Daire V, Beyler C, Manach Y, Munnich A, et al. Pierre Robin sequence: a series of 117 consecutive cases. J Pediatr. 2001;139(4):588-90.
117. Khoshhal K, Letts RM. Orthopaedic manifestations of campomelic dysplasia. Clin Orthop Relat Res. 2002(401):65-74.
118. Argentaro A, Sim H, Kelly S, Preiss S, Clayton A, Jans DA, et al. A SOX9 defect of calmodulin-dependent nuclear import in campomelic dysplasia/autosomal sex reversal. J Biol Chem. 2003;278(36):33839-47.
119. Savarirayan R, Robertson SP, Bankier A, Rogers JG. Variable expression of campomelic dysplasia in a father and his 46, XY daughter. Pediatr Pathol Mol Med. 2003;22(1):37-46.
120. Üner Ç, Ekşioǧlu AS. Campomelic dysplasia: A long surviving patient with sex reversal. Gazi Medical Journal. 2003;14(3):131-4.
121. Cormier-Daire V, Geneviève D, Munnich A, Le Merrer M. New insights in congenital bowing of the femora. Clin Genet. 2004;66(3):169-76.
122. Jamshidi N, Macciocca I, Dargaville PA, Thomas P, Kilpatrick N, McKinlay Gardner RJ, et al. Isolated Robin sequence associated with a balanced t(2;17) chromosomal translocation. J Med Genet. 2004;41(1):e1.
123. Seow KM, Huang LW, Lin YH, Pan HS, Tsai YL, Hwang JL. Prenatal three-dimensional ultrasound diagnosis of a camptomelic dysplasia. Arch Gynecol Obstet. 2004;269(2):142-4.
124. Lekovic GP, Rekate HL, Dickman CA, Pearson M. Congenital cervical instability in a patient with camptomelic dysplasia. Childs Nerv Syst. 2006;22(9):1212-4.
125. Natasha G, Ghai R, Shah D, Kiran PS. Camptomelic dysplasia: prenatal diagnosis by ultrasound. Skeletal Radiol. 2006;35(9):699-701.
126. Promsonthi P, Wattanasirichaigoon D. Prenatal diagnosis of campomelic dysplasia with three-dimensional ultrasound. Ultrasound Obstet Gynecol. 2006;27(5):583-5.
127. Zody MC, Garber M, Adams DJ, Sharpe T, Harrow J, Lupski JR, et al. DNA sequence of human chromosome 17 and analysis of rearrangement in the human lineage. Nature. 2006;440(7087):1045-9.
128. Kos R, Medjo B, Grković S, Nikolić D, Sajić S, Ilić J. [Camptomelic dysplasia--a case report]. Srp Arh Celok Lek. 2007;135(5-6):335-8.
129. Gimovsky M, Rosa E, Tolbert T, Guzman G, Nazir M, Koscica K. Campomelic dysplasia: case report and review. J Perinatol. 2008;28(1):71-3.
130. Gordon CT, Tan TY, Benko S, Fitzpatrick D, Lyonnet S, Farlie PG. Long-range regulation at the SOX9 locus in development and disease. J Med Genet. 2009;46(10):649-56.
131. Coban D, Akin MA, Ozturk MA, Gunes T, Secilmis Y. Campomelic dysplasia: Presentation of a newborn. Early Human Development. 2010;86:S95.
132. Courtens W, Desmyter L, Bayetand B, Vikkula M. A child and his mother with Pierre Robin syndrome caused by a microdeletion in the long arm of chromosome 17 (17q24.3). Genetic Counseling. 2010;21(1):140-1.
133. Dahdaleh NS, Albert GW, Hasan DM. Campomelic dysplasia: a rare cause of congenital spinal deformity. J Clin Neurosci. 2010;17(5):664-6.
134. Refai O, Friedman A, Terry L, Jewett T, Pearlman A, Perle MA, et al. De novo 12;17 translocation upstream of SOX9 resulting in 46,XX testicular disorder of sex development. Am J Med Genet A. 2010;152a(2):422-6.
135. Barros A, Teixeira F, Camacho MC, Alves C. Campomelic dysplasia and malignant hyperthermia. BMJ Case Rep. 2011;2011.
136. Benko S, Gordon CT, Mallet D, Sreenivasan R, Thauvin-Robinet C, Brendehaug A, et al. Disruption of a long distance regulatory region upstream of SOX9 in isolated disorders of sex development. J Med Genet. 2011;48(12):825-30.
137. Capito C, Leclair MD, Arnaud A, David A, Baron S, Corradini N, et al. 46,XY pure gonadal dysgenesis: clinical presentations and management of the tumor risk. J Pediatr Urol. 2011;7(1):72-5.
138. Cox JJ, Willatt L, Homfray T, Woods CG. A SOX9 duplication and familial 46,XX developmental testicular disorder. New England Journal of Medicine. 2011;364(1):91-3.
139. White S, Ohnesorg T, Notini A, Roeszler K, Hewitt J, Daggag H, et al. Copy number variation in patients with disorders of sex development due to 46,XY gonadal dysgenesis. PLoS One. 2011;6(3):e17793.
140. Amarillo IE, Dipple KM, Quintero-Rivera F. Familial microdeletion of 17q24.3 upstream of SOX9 is associated with isolated Pierre Robin sequence due to position effect. Am J Med Genet A. 2013;161a(5):1167-72.
141. Fanelli M, Silva RB, Domenice S, Mendonca BB, Costa EMF. Analysis of Sox9 gene expression regulatory region in 46,XY DSD patients without campomelic dysplasia. Hormone Research in Paediatrics. 2013;80:185.
142. Sarvestani RT, Fatholapour A, Gharib A. Camptomelic dysplasia in a 38 weeks neonate - A rare case report. Life Science Journal. 2013;10(SPEC. ISSUE 9):277-9.
143. Scherer G, Zabel B, Nishimura G. Clinical Utility Gene Card for: campomelic dysplasia. Eur J Hum Genet. 2013;21(7).
144. Smyk M, Szafranski P, Startek M, Gambin A, Stankiewicz P. Chromosome conformation capture-on-chip analysis of long-range cis-interactions of the SOX9 promoter. Chromosome Res. 2013;21(8):781-8.
145. Gordon CT, Attanasio C, Bhatia S, Benko S, Ansari M, Tan TY, et al. Identification of novel craniofacial regulatory domains located far upstream of SOX9 and disrupted in Pierre Robin sequence. Hum Mutat. 2014;35(8):1011-20.
146. Jain V, Sen B. Campomelic dysplasia. J Pediatr Orthop B. 2014;23(5):485-8.
147. Kertikova T, Valentinov B, Arabadzliieva D, Nedeva T, Petrov V. Anesthesia in rare diseases-pathologic femur fracture in a child with campomelic dysplasia. Anaesthesiology and Intensive Care. 2014;43(4):44-6.
148. Pickart A, Chirempes B, Wittke J, Virlee E, Scharer G. 5.3 megabase chromosome 17q24.2q25.1 deletion associated with SOX9 haploinsufficiency in a 46, XX female presenting with an acampomelic phenotype. Journal of Molecular Diagnostics. 2014;16(6):700.
149. Kim GJ, Sock E, Buchberger A, Just W, Denzer F, Hoepffner W, et al. Copy number variation of two separate regulatory regions upstream of SOX9 causes isolated 46,XY or 46,XX disorder of sex development. Journal of Medical Genetics. 2015;52(4):240-7.
150. Smyk M, Roeder E, Cheung SW, Szafranski P, Stankiewicz P. A de novo 1.58 Mb deletion, including MAP2K6 and mapping 1.28 Mb upstream to SOX9, identified in a patient with Pierre Robin sequence and osteopenia with multiple fractures. Am J Med Genet A. 2015;167a(8):1842-50.
151. Lefebvre V. Roles and regulation of SOX transcription factors in skeletogenesis. Current topics in developmental biology. 2019;133:171-93.
152. Narimatsu K, Iida A, Kobayashi T. Palatoplasty for the Patient With Campomelic Dysplasia-Report of a Case and Review of the Literature. Cleft Palate Craniofac J. 2022;59(1):132-6.
153. Ježová M, Pavlovská D, Grochová I, Michenková A, Vlašín P. Skeletal dysplasias of the fetus and infant: comprehensive review and our experience over a 10-year period. Cesk Patol. 2023;59(2):68-79.
